# Gov. Cleveland and the Homœopaths

**Published:** 1884-10-20

**Authors:** 


### GOV. CLEVELAND AND THE HOMŒOPATHS.

Our homœopathic friend of the New York Medical Times complains that Governor Cleveland, in his vetoes, has discriminated in favor of the Allopaths. It is probable, however, that his action was on other grounds than that of preference for any particular school of medicine, as he is known to be a fair, cautious and strictly honest man.

It is said of Mr. Lincoln, however, that he was violently opposed to homœopathy, and on one occasion rejected an application for a place on the medical staff in a way to humiliate the party applying for the position by telling one of his characteristic anecdotes, in which he illustrated his opinion of infinitesimal medicine in a strong and ridiculous light.

